# *SOCS3* Promotes ALV-J Virus Replication *via* Inhibiting JAK2/STAT3 Phosphorylation During Infection

**DOI:** 10.3389/fcimb.2021.748795

**Published:** 2021-09-10

**Authors:** Guodong Mo, Huali Fu, Bowen Hu, Qihong Zhang, Mingjian Xian, Zihao Zhang, Ling Lin, Meiqing Shi, Qinghua Nie, Xiquan Zhang

**Affiliations:** ^1^Department of Animal Genetics, Breeding and Reproduction, College of Animal Science, South China Agricultural University, Guangzhou, China; ^2^Guangdong Provincial Key Lab of AgroAnimal Genomics and Molecular Breeding and Key Lab of Chicken Genetics, Breeding and Reproduction, Ministry of Agriculture and Rural Affairs, Guangzhou, China; ^3^Division of Immunology, Virginia-Maryland Regional College of Veterinary Medicine, University of Maryland, College Park, MD, United States

**Keywords:** ALV-J, chicken, immune, JAK2/STAT3, *SOCS3*

## Abstract

Avian leukosis virus subgroup J (ALV-J) is an oncogenic retrovirus that causes immunosuppression and neoplastic diseases in poultry. Cytokine signal-transduction inhibitor molecule 3 (SOCS3) is an important negative regulator of the JAK2/STAT3 signaling pathway and plays certain roles in ALV-J infection. It is of significance to confirm the roles of *SOCS3* in ALV-J infection and study how this gene affects ALV-J infection. In this study, we assessed the expression of the *SOCS3* gene *in vivo* and *in vitro*, and investigated the roles of *SOCS3* in ALV-J infection using overexpressed or interfered assays with the *SOCS3* in DF-1 cells. The results showed that the *SOCS3* expression of ALV-J infected chickens was different from uninfected chickens in the spleen, thymus and cecal tonsil. Further, *SOCS3* is mainly expressed in the nucleus as determined by immunofluorescence assay. Overexpression of *SOCS3* in DF-1 cells promoted the replication of ALV-J virus, and the expression of interferons (*IFNα* and *INFβ*), inflammatory factors (*IL-6* and *TNFα*) along with interferon-stimulating genes (*CH25H*, *MX1*, *OASL*, and *ZAP*). Conversely, interference of *SOCS3* showed the opposite results. We also observed that SOCS3 promoted ALV-J virus replication by inhibiting JAK2/STAT3 phosphorylation. In conclusion, *SOCS3* promotes ALV-J replication *via* inhibiting the phosphorylation of the JAK2/STAT3 signaling pathway. These results would advance further understanding of the persistent infection and the viral immune evasion of the ALV-J virus.

## Introduction

Avian leukemia virus subgroup J was first isolated and identified from commercial broilers in 1988, which mainly causes myeloid leukosis (Payne et al., 1991; Payne et al., 1992). Infection with ALV-J causes immunosuppression and decreases chickens production performance, leading to serious economic losses in the poultry industry. ALV-J virus can transmit vertically and horizontally, and the viral genome is highly variable. There are no treatments or vaccines to prevent ALV-J infection, and eradication of ALV-J can be achieved only by chicken population cleansing (Payne and Nair, 2012). Therefore, it is necessary to study the immune response mechanisms of hosts after ALV-J virus infection.

Previously, we performed RNA-seq on chicken primary monocyte-derived macrophages (MDM) cells infected with ALV-J. The results revealed that the cytokine signal transduction inhibitor 3 (*SOCS3*) was a differentially expressed gene in the JAK/STAT signaling pathway (Feng et al., 2019). Also, we further found that the *SOCS3* gene was one of the differentially expressed genes based on the RNA-seq data (PRJNA552417) of spleen tissues from 7 days-old chicken infected with ALV-J (data unpublished). Furthermore, overexpression of *SOCS3* in MDM cells promotes ALV-J virus replication (Feng et al., 2019). These observations imply that *SOCS3* may play an important role in ALV-J virus replication in chickens.

SOCS3 is an inhibitor of signaling pathways initiating cytokines, which belongs to the SOCS family (Chen et al., 2000). It is mainly involved in the negative feedback regulation of the tyrosine-protein kinase/signal transduction pathway and transcriptional activator signal transduction pathway. Furthermore, SOCS3 is closely related to inflammatory response, oxidative stress, cell injury, and apoptosis (Nicholson et al., 2000; Rawlings et al., 2004; Kershaw et al., 2013). The critical role of SOCS3 is manifested by its binding to both the JAK kinase and the cytokine receptor, which further inhibits STAT3 phosphorylation (Harrison, 2012). The JAK/STAT pathway is an evolutionarily conserved signaling pathway that transduces signals from extracellular to the nucleus (Rawlings et al., 2004). JAK/STAT pathway activation stimulates cell proliferation, differentiation, cell migration, and immune challenge (Rawlings et al., 2004; Harrison, 2012; Coskun et al., 2013). The JAK/STATs signaling pathway consists of three main components: 1. tyrosine kinase associated receptor; 2. JAK kinases; 3. STAT proteins (Gao et al., 2018). In recent years, many studies have reported that the JAK/STAT signaling pathway plays an important role in viral infection (Cherng et al., 2021; Leite et al., 2021; Wang et al., 2021; Ye et al., 2021).

Newcastle disease virus (NDV) infection activates the expression of SOCS3 at the mRNA and protein level through a mechanism that depends on the MEK/ERK signaling pathway, which is conducive to the virus replication (Wang et al., 2019a). Similarly, overexpression of *SOCS3* in DF-1 cells promotes infectious bursal disease (IBD) virus replication (Duan et al., 2020). Porcine reproductive and respiratory syndrome virus (PRRSV) infection also induces *SOCS3* expression through p38/AP-1 signaling pathway to enhance PRRSV replication during infection (Luo et al., 2021). In addition, SOCS3 also plays an important role in bacterial infections, inflammatory responses, and cancer (Gao et al., 2018; Fukuta et al., 2021; Matsumura et al., 2021). However, the mechanism of *SOCS3* on ALV-J virus replication remains unclear. Thus, in this study, we focused on analyzing the expression of *SOCS3* after ALV-J infection and the mechanism of effects of *SOCS3* on ALV-J replication.

## Materials and Methods

### Ethics Statement

All animal experiments in this study were conducted following the protocols approved by the Institutional Animal Care and Use Committee of South China Agriculture University (No: SCAU 2018c008) and following the Animal Protection Law of the People’s Republic of China.

### Tissue Samples Source

The 280 days-old chickens were from a local farm in Guangdong. They showed telltale symptoms of infection, such as a pale cockscomb, listlessness, a thin body, and obvious hemangiomas on their skin and digits. Zhang et al. (2021) have verified that the chickens were only infected with the ALV-J virus. We collected the spleen, thymus, and cecal tonsils from chickens infected with ALV-J (n = 3) and uninfected with ALV-J (n = 3), and stored them at −80°C till their later use.

### Virus and Cells

The laboratory ALV-J strain SCAU-HN06 was kindly provided by Prof. Weisheng Cao (South China Agricultural University, Guangzhou, China). The ALV-J strain SCAU-HN06 was isolated from commercial layer hens with spontaneous hemangiomas in China (Zhang et al., 2011). Chicken embryo fibroblast (DF-1) cells were obtained from ATCC (Manassas, VA, USA) and were maintained in Dulbecco’s modified Eagle’s medium (DMEM) (Gibco, USA) supplemented with 10% fetal bovine serum (FBS, Hyclone, USA) and 0.1% penicillin/streptomycin (Invitrogen, USA).

### Overexpression and siRNA Knockdown Assay

The *SOCS3* gene of chicken (GenBank ID. NM_204600) was cloned into a pcDNA3.1 vector (Invitrogen) with the primers sequences to generate the vector named pcDNA3.1-*SOCS3*. The forward primer sequence (5′ - 3′): ***CCGGAAT***TCATGGTCACCCACAGCAAG and the reverse sequence (5′-3′): ***TGCTCTA***GAGATTTCCCTCTGCCAGCCT was used to generate the pcDNA3.1-SOCS3 vector. ***Blue*** letters represent enzyme cutting sites. In our preliminary experiments, we designed three siRNAs for each gene to interfere with SOCS3, JAK2, STAT3 and the siRNA with the highest interference efficiency was chosen. The results of knockdown and overexpression efficiency are show in **Supplementary Figure S1**. The specific small interfering RNA (siRNAs) were designed and constructed by Genewiz (Nanjing, China), and the sequences of siRNAs were as follows: si-*SOCS3* 5′- GCUUCUACUGGAGCACGGUTT-3′, si-*JAK2* 5′-GTCATGTCTTACCTATTTG-3′, si-*STAT3* 5′-GGACATCAGTGGAAAGACT-3′, a scrambled negative control RNA (si-NC) 5′-UUCUCCGAACGUGUCACGUTT-3′.

A total of 1 × 10^5^ DF-1 cells/well were cultured in DMEM with 10% FBS and 0.1% penicillin/streptomycin overnight. According to the manufacturer’s instructions, the DF-1 cells were transfected with pcDNA3.1-SOCS3 plasmid or siRNAs by Lipofectamine 3000 reagent (Invitrogen, USA). pcDNA3.1 or si-NC was used as a control, respectively. After 24 h transfection, the DF-1 cells were infected with ALV-J strain SCAU-HN06 (10^5^ TCID_50_/mL). Total protein, RNA, and cell supernatants samples were collected at 24 h and 48 h post-infection (hpi). Subsequently, qRT-PCR, ELISA, or western blotting (WB) were used to assess the roles of *SOCS3* in ALV-J infection.

### RNA Isolation and cDNA Synthesis

Following the manufacturer’s protocol, total RNA was extracted from tissues and DF-1 cells using the RNAiso reagent (Takara, Japan). RNA integrity and concentration were determined using 1% agarose gel electrophoresis and a Nanodrop 2000c spectrophotometer (Thermo, USA). cDNA was synthesized using MonAmp™ RTIII All-in-One Mix (Monad, Guangzhou, China), following the manufacturer′s protocol. Synthesized cDNA was stored at -20°C until subsequent analysis using qRT-PCR.

### Quantitative Real-Time PCR

qRT-PCR primers specific for *SOCS3*, *JAK2*, *STAT3*, *IFNα*, *IFNβ*, *IL-6*, *TNFα*, *ZAP*, *CH25H*, *MX1*, and *OASL* were designed using Oligo 7.0 software. All primers were synthesized by Tsingke Biotech Technology Co., Ltd. (Guangzhou, China). The MonAmp™ SYBR^®^ Green qPCR Mix (Monad, Guangzhou, China) was used for qPCR in an ABI 7500 Real-Time Detection instrument (Applied Biosystems, USA) following the manufacturer’s protocol. Relative gene expression was measured by qRT-PCR three for each reaction, and the nuclear GAPDH gene was used as a control. The *gp85* of ALV-J virus copy was performed as previously described (Dai et al., 2015). The primers used in qRT-PCR were shown in **Table S1**.

### Immunofluorescence Assay

Immunofluorescence assay was performed as previously described (Zhang et al., 2020). Rabbit anti-SOCS3 antibodies were used (XY-10R-5878; Fitzgerald, USA; 1:500) and were incubated at 4° for 8 h. The goat anti-rabbit IgG H&L/FITC (bs-0295G-FITC; Bioss, China; 1:500) was used to combine the antibody/antigen complex at room temperature for 1 h. The nuclei were dyed by 4′,6-diamidino-2-phenylindole (DAPI) for 5 min, and a fluorescence microscope (Nikon, Tokyo, Japan) was utilized to capture the immunofluorescence pictures.

### Western Blotting Assay

Western Blotting (WB) assays were performed as previously described (Zhang et al., 2020). The antibodies and their dilutions used for WB were as follows: anti-ALV-J envelope protein-specific monoclonal antibody JE9 (kindly provided by Prof. Aijian Qin, Yangzhou University, 1:1000), Rabbit Anti-JAK2 antibody (bs-0908R; Boss, China; 1:1000), Rabbit Anti-phospho-JAK2 (Tyr1007+Tyr1008) (bsm-52171R; Boss, China; 1:1000), Rabbit Anti-STAT3 antibody (bs-1141R; Boss, China; 1:1000), Rabbit Anti-phospho-STAT3 (Ser727) antibody (bs-3429R; Boss, China; 1:1000), Rabbit Anti-beta-Actin antibody (bs-0061R; Boss, China; 1:1000), Goat Anti-Rabbit IgG H&L/HRP antibody (bs-40295G-HRP; Boss, China; 1:5000).

### ELISA

According to the manufacturer’s protocol, the p27 (the main protein component of the ALV viral capsid) level was measured using an avian leukosis virus antigen test kit (IDEXX, USA). The IFNα, IFNβ, IL-6, and TNFα levels were measured using a Chicken Interferon α (IFNα) ELISA Kit (ML760024; mlbio, Shanghai, China), Chicken Interferon β (IFNβ) ELISA Kit (ML760024; mlbio, Shanghai, China), Chicken Interleukin 6 (IL-6) ELISA Kit (ML760041; mlbio, Shanghai, China), and Chicken Tumor necrosis factor α (TNFα) ELISA Kit (ML760161; mlbio, Shanghai, China) according to the manufacturer′s protocol, respectively. In brief, cells and medium were collected from tissue culture dishes, subjected to three freeze-thaw cycles, and centrifuged at 3000 × *g* at 4°C for 10 min. The supernatants were collected and subjected to ELISA according to the manufacturer′s protocols.

### Statistical Analysis

Statistical comparisons were performed using GraphPad Prism 5 (GraphPad Software Inc., La Jolla, CA, USA). The data are presented as means ± standard error of the mean (SEM). The date differences in data were evaluated by the Student′s *t*-test. A *P* value of < 0.05 was considered statistically significant (*P ≤ 0.05, **P ≤ 0.01, and ***P ≤ 0.001).

## Results

### The Expression of *SOCS3* in Immune Organs and DF-1 cells

**Figure 1:**
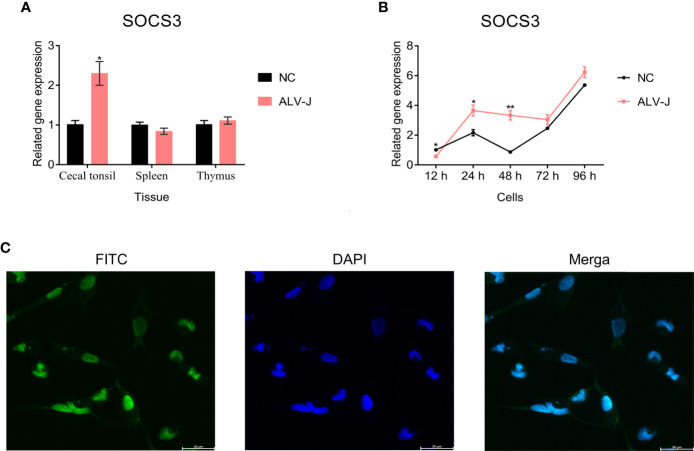
The expression of *SOCS3* in tissues and cells. **(A)** The expression of *SOCS3* in immune organs of ALV-J infected chickens (n = 3) and uninfected chickens (n = 3) was quantified by qRT-PCR. The chickens were only infected with ALV-J, as previously demonstrated by Zhang et al. (2021). **(B)** After DF-1 cells were infected with 10^5^ TCID_50_/mL ALV-J strain SCAU-HN06, the expression of *SOCS3* in cells was measured from 12 hpi to 96 hpi by qRT-PCR. **(C)** Immunofluorescence analysis of SOCS3 in DF-1 cells. Scale bar: 20 μm. A special antibody against SOCS3 was modified by FITC (green). The nucleus was stained by DAPI (blue). These experiments were performed independently at least three times independently. Differences in data were evaluated by the Student’s *t*-test. The error bars are the standard error of the mean (SEMs) (*P ≤ 0.05, and **P ≤ 0.01).

First, we evaluated the expression of *SOCS3* in the spleen, thymus, and cecal tonsil of ALV-J infected and uninfected chickens by qRT-PCR. The chickens were only infected with ALV-J, as previously demonstrated by Zhang et al. (2021). The expression of *SOCS3* in the cecal tonsil (*P* < 0.05) and thymus (*P* > 0.05) of ALV-J infected individuals was higher than that of uninfected individuals (**Figure 1A**). Next, we collected and detected DF-1 cells after ALV-J infection at different time points to investigate the *SOCS3* expression *in vitro*. The expression of *SOCS3* in DF-1 cells infected with ALV-J was higher than that of uninfected cells, except at 12 h post-infection (hpi) (**Figure 1B**). Besides, immunofluorescence assay results showed that *SOCS3* was expressed in the nucleus and cytoplasm, but mainly in the nucleus (**Figure 1C**).

### Overexpression and Knockdown of *SOCS3* Could Influence ALV-J Virus Replication

**Figure 2:**
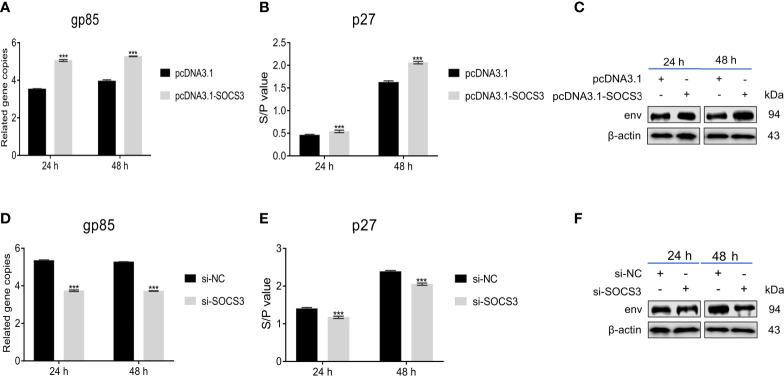
Overexpression and knockdown of *SOCS3* in DF-1 cells could influence ALV-J virus replication. After 24 h of overexpression of *SOCS3* in DF-1 cells, cells were infected with 10^5^ TCID_50_/mL ALV-J strain SCAU-HN06. Samples were collected at 24 and 48 hpi, and qRT-PCR **(A)**, ELISA **(B)**, and WB **(C)** were performed for ALV-J virus content. After 24 h knockdown of *SOCS3*, DF-1 cells were infected with 10^5^ TCID_50_/mL ALV-J strain SCAU-HN06. Samples were collected at 24 and 48 hpi, and qRT-PCR **(D)**, ELISA **(E)**, and WB **(F)** were performed for ALV-J virus content. These experiments were performed at least three times independently with similar results. Differences in data were evaluated by the Student’s *t*-test *t* test. The error bars are the standard error of the mean (SEMs) (***P ≤ 0.01).

To explore the functions of *SOCS3*, we overexpressed and interfered with *SOCS3* in DF-1 cells to evaluate its effect on ALV-J virus replication. The gp85, the major viral envelope protein, is the most variable of the structural proteins in the genome of ALV-J and exhibits high diversity (Venugopal et al., 1998; Pan et al., 2012). The capsid protein ALV p27 is encoded by the gag gene and acts as a major group-specific antigen (Weiss, 2006; Yun et al., 2013). Therefore, we evaluate the effect of SOCS3 on ALV-J virus replication by qRT-PCR (gp85) and ELISA (p27). The qRT-PCR, ELISA, and WB results showed that the overexpression of *SOCS3* in DF-1 cells promoted the expression of gp85 and p27, suggesting that the *SOCS3* benefits the replication of ALV-J virus (**Figures 2A–C**). In contrast, knockdown of *SOCS3* in DF-1 cells inhibited the ALV-J virus replication (**Figures 2D–F**).

### SOCS3 Affects the Phosphorylation of the JAK2 and STAT3

**Figure 3:**
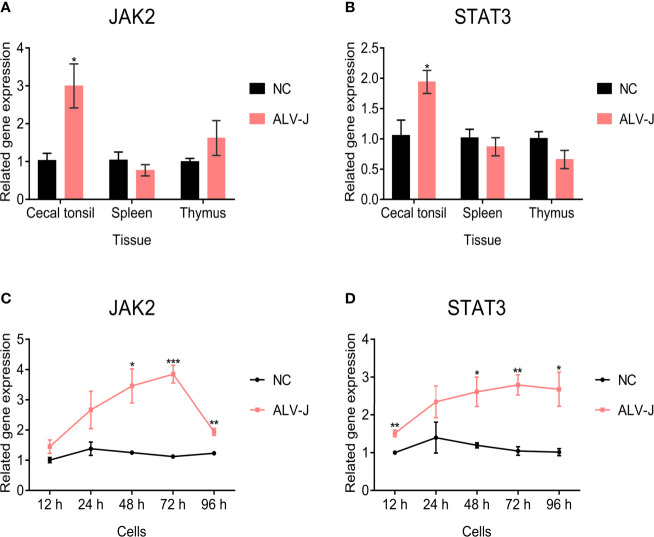
JAK2 and STAT3 gene expression in tissues and cells. The **(A)** *JAK2* and **(B)** *STAT3* expression in immune organs of ALV-J infected chickens (n = 3) and uninfected chickens (n = 3) were quantified by qRT-PCR. The chickens were only infected with ALV-J, as previously demonstrated by Zhang et al. (2021). After DF-1 cells were infected with ALV-J strain SCAU-HN06, the **(C)** *JAK2* and **(D)** *STAT3* genes expression was detected by qRT-PCR from 12 hpi to 96 hpi. These experiments were performed independently at least three times with similar results. Differences in data were evaluated by the Student’s *t*-test. The error bars are the standard error of the mean (SEMs) (*P ≤ 0.05, **P ≤ 0.01, and ***P ≤ 0.001).

Since SOCS3 plays an important role *via* the JAK/STAT signaling pathway (Chen et al., 2000; Rawlings et al., 2004), we further evaluated the expression of *JAK2* and *STAT3* genes in ALV-J infected and uninfected tissues and cells to study how SOCS3 promotes ALV-J virus replication. The expression of *JAK2* was higher in the cecal tonsil but not in the thymus, since the difference was not significant in the thymus after ALV-J infection compared with uninfected chickens. In contrast, the expression of *JAK2* in the spleen was lower than in the uninfected chickens (**Figure 3A**). The expression of *STAT3* was higher in the cecal tonsil but lower in the spleens and thymus in chickens infected with ALV-J, compared to uninfected chickens (**Figure 3B**). Furthermore, after the DF-1 cells were infected with ALV-J, the expression of *JAK2* and *STAT3* were increased and higher than that of uninfected cells from 12 hpi to 96 hpi (**Figures 3C, D**).

**Figure 4:**
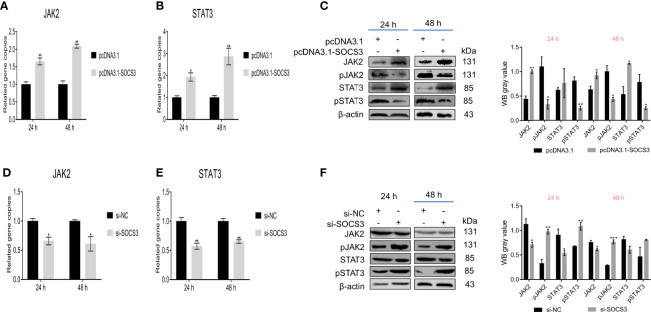
SOCS3 affects JAK2 and STAT3 phosphorylation. After overexpression of *SOCS3* in DF-1 cells, the *JAK2* and *STAT3* expression and the phosphorylation levels were detected by qRT-PCR **(A, B)** and WB **(C)**. After the knockdown of *SOCS3* in DF-1 cells, the *JAK2* and *STAT3* expression and the phosphorylation levels were detected by qRT-PCR **(D, E)** and WB **(F)**. These experiments were performed independently at least three times with similar results. Differences in data were evaluated by the Student’s *t*-test. The error bars are the standard error of the mean (SEMs) (*P ≤ 0.05, **P ≤ 0.01, and ***P ≤ 0.001).

Based on the above results, we speculated that SOCS3 might play an important role in ALV-J infection through JAK2/STAT3. Therefore, we analyzed the effect of SOCS3 on the JAK2/STAT3 signaling pathway. Overexpression of *SOCS3* in DF-1 cells significantly increased the *JAK2* and *STAT3* mRNA expression, and significantly decreased the phosphorylation levels of JAK2 and STAT3 (**Figures 4A–C**). In contrast, knockdown of *SOCS3* in DF-1 cells significantly decreased the *JAK2* and *STAT3* mRNA expression and significantly increased the phosphorylation levels of JAK2 and STAT3 (**Figures 4D–F**).

**Figure 5:**
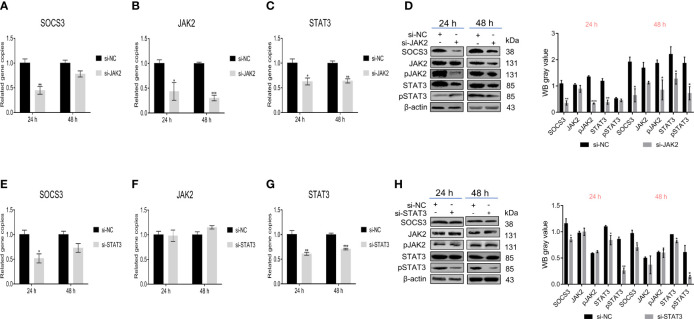
JAK2/STAT3 inhibited SOCS3 expression. After the knockdown of *JAK2* in DF-1 cells, the *SOCS3*, *JAK2*, and *STAT3* genes expression and the phosphorylation levels of JAK and STAT3 were detected by qRT-PCR **(A–C)** and WB **(D)**. After knockdown of *STAT3* in DF-1 cells, the *SOCS3* and *JAK2* genes expression and the phosphorylation levels of JAK and STAT3 were detected by qRT-PCR **(E–G)** and WB **(H)**. These experiments were performed independently at least three times with similar results. Differences in data were evaluated by the Student’s *t*-test. The error bars are the standard error of the mean (SEMs) (*P ≤ 0.05, **P ≤ 0.01, and ***P ≤ 0.001).

Besides, we further verified the effect of JAK2/STAT3 on *SOCS3*. Knockdown of *JAK2* in DF-1 cells significantly reduced the *SOCS*3 and *STAT3* expression and the phosphorylation levels of JAK2 and STAT3 (**Figures 5A–D**). Also, knockdown of *STAT3* in DF-1 cells significantly reduced the *SOCS3* expression and the phosphorylation of STAT3, but did not affect the *JAK2* expression and phosphorylation of JAK2 (**Figures 5E–H**). The above experimental data demonstrated that *SOCS3* is a negative regulator of the JAK2/STAT3 signaling pathway.

### SOCS3 Affects ALV-J Virus Replication by Inhibiting JAK2/STAT3 Phosphorylation

**Figure 6:**
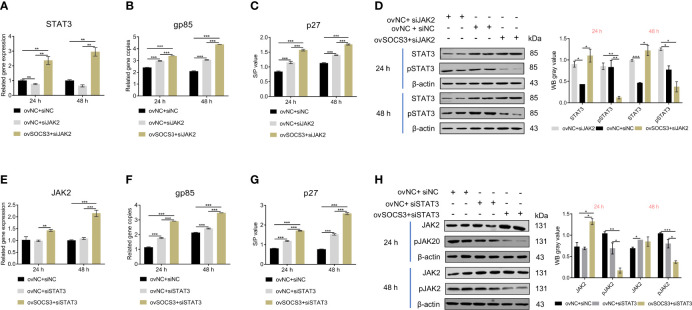
The effects of JAK2/STAT3 phosphorylation on ALV-J virus replication. The pcDNA3.1-*SOCS3* and si-*JAK2* were co-transfected into DF-1 cells. The transfected cells were infected with 10^5^ TCID_50_/mL ALV-J strain SCAU-HN06 after 24 h. The **(A)** *STAT3* gene and **(B)** ALV-J virus were detected by qRT-PCR. The **(C)** ALV-J virus (p27) and **(D)** STAT3 phosphorylation were detected by ELISA and WB, respectively. The pcDNA3.1-*SOCS3* and si-*STAT3* were co-transfected into DF-1 cells. The transfected cells were infected with 10^5^ TCID_50_/mL ALV-J strain SCAU-HN06 after 24 h. The **(E)** *STAT3* gene and **(F)** ALV-J virus were detected by qRT-PCR. The **(G)** ALV-J virus (p27) and **(H)** STAT3 phosphorylation were detected by ELISA and WB, respectively. pcDNA3.1+si-NC, ovNC + siNC; pcDNA3.1+si-*JAK2*, ovNC + si-*JAK2*; pcDNA3.1-*SOCS3* + si-*JAK2*, ovSOCS3 + siJAK2; pcDNA3.1+si-*STAT3*, ovNC + si-*STAT3*; pcDNA3.1-*SOCS3* + si-*STAT3*, ovSOCS3 + si *STAT3*. These experiments were performed independently at least three times with similar results. Differences in data were evaluated by the Student’s *t*-test. The error bars are the standard error of the mean (SEMs) (*P ≤ 0.05, **P ≤ 0.01, and ***P ≤ 0.001).

To investigate how *SOCS3* affects ALV-J virus replication, we conducted co-transfection experiments with pcDNA3.1-*SOCS3*, si-*JAK2*, and si-*STAT3*, respectively. After the knockdown of *JAK2*, the *STAT3* expression decreased while the ALV-J virus content increased compared to the control. In pcDNA3.1-*SOCS3* + si-*JAK2* groups, the *STAT3* expression was significantly increased, but the phosphorylation level of STAT3 was decreased. Both the qRT-PCR and ELISA results showed that the content of the ALV-J virus was significantly increased (**Figures 6A–D**). After the knockdown of *STAT3*, there was no significant difference in *JAK2* expression, but the ALV-J virus’s content significantly increased compared to the control. In pcDNA3.1-*SOCS3* + si-*STAT3* groups, the *STAT3* expression and ALV-J virus were significantly increased, but the phosphorylation level of JAK2 was decreased (**Figures 6E–H**). These results above indicated that *SOCS3* inhibits the phosphorylation of JAK2 and STAT3, thereby affecting the replication of the ALV-J virus.

### SOCS3 Affects the Expression of Interferon, Inflammatory Factors, and Interferon-Stimulated Genes

**Figure 7:**
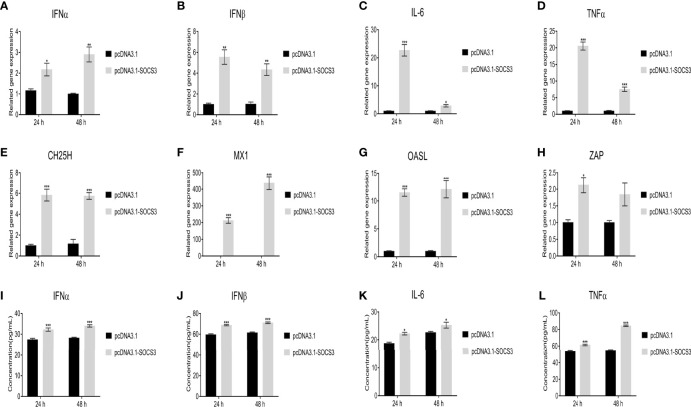
Overexpression of *SOCS3* promotes the expression of interferons, inflammatory factors, and interferon-stimulated genes. After *SOCS3* overexpression in DF-1 cells in 24h, the cells were infected with 10^5^ TCID_50_/mL ALV-J strain SCAU-HN06. At 24 and 48 hpi, qRT-PCR was used to detect **(A)** *IFNα*, **(B)** *IFNβ*, **(C)** *IL-6*, **(D)** *TNFα*, **(E)** *CH25H*, **(F)** *MX1*, **(G)** *OASL*, and **(H)** *ZAP* mRNA levels. After three freeze-thaw cycles, the levels of the content of **(I)** IFNα, **(J)** IFNβ, **(K)** IL-6, and **(L)** TNFα in cells were detected by ELISA assay. These experiments were performed independently at least three times with similar results. Differences in data were evaluated by the Student’s *t*-test. The error bars are the standard error of the mean (SEMs) (*P ≤ 0.05, **P ≤ 0.01, and ***P ≤ 0.001).

**Figure 8:**
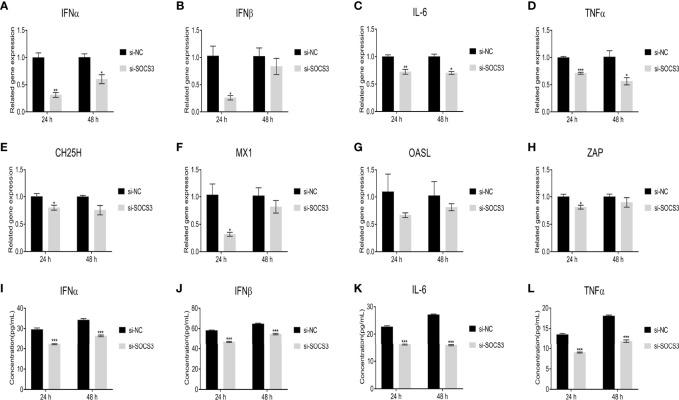
Knockdown of *SOCS3* reduces the expression of interferons, inflammatory factors, and interferon-stimulated genes. *SOCS3* knockdown in DF-1 cells in 24h, the cells were infected with 10^5^ TCID_50_/mL ALV-J strain SCAU-HN06. At 24 and 48 hpi, qRT-PCR was used to detect **(A)** *IFNα*, **(B)** *IFNβ*, **(C)** *IL-6*, **(D)** *TNFα*, **(E)** *CH25H*, **(F)** *MX1*, **(G)** *OASL*, and **(H)** *ZAP* mRNA levels. After three freeze-thaw cycles, the levels of the content of **(I)** IFNα, **(J)** IFNβ, **(K)** IL-6, and **(L)** TNFα in cells were detected by ELISA assay. These experiments were performed independently at least three times with similar results. Differences in data were evaluated by the Student’s *t*-test. The error bars are the standard error of the mean (SEMs) (*P ≤ 0.05, **P ≤ 0.01, and ***P ≤ 0.001).

In the present study, the ALV-J virus significantly increased the mRNA expression levels of *IFNα*, *IFNβ*, *IL-6*, and *TNFα* after overexpression of *SOCS3* in DF-1 cells (**Figures 7A–D**). Furthermore, overexpression of *SOCS3* significantly promoted the mRNA expression of interferon-stimulated genes *ZAP*, *CH25H*, *Mx1*, and *OASL* (**Figures 7E–H**). After three freeze-thaw cycles, DF-1 cells were detected by ELISA. The results were similar to those of qRT-PCR. Overexpression of *SOCS3* significantly increased the content of IFNα, IFNβ, IL-6, and TNFα (**Figures 7I–L**). However, the expression of *IFNα*, *IFNβ*, *IL-6*, *TNFα*, *ZAP*, *CH25H*, *MX1*, and *OASL* genes significantly decreased after SOCS knockdown in DF-1 cells (**Figure 8**). It may be associated with ALV-J persistent infection.

## Discussion

Although researchers have learned a great deal about innate and adaptive immunity, several important questions need to be addressed. The virus could develop some strategies to escape the host immune responses. It can affect virus replication by neutralizing antibody (NAb) escape mutants (Pandiri et al., 2010) or by inhibiting/promoting the expression of certain genes (Feng et al., 2019). Some genes help the virus survive in the host; for example, the JAK/STAT pathway inhibitors (*CISH*, *SOCS1*, and *SOCS3*) are beneficial to ALV-J survival in MDM cells (Feng et al., 2019). In our previous data (RNA-seq, PRJNA552417), *SOCS3* was highly expressed in slow-feathered chickens compared to late-feathered chickens (unpublished). Evidence shows that slow-feathered chickens are susceptible to ALV-J (Bacon et al., 1988; Fadly and Smith, 1997). These above results suggest that *SOCS3* may play an important role in ALV-J virus infection.

Phosphotyrosine phosphatases (PTPs), protein inhibitors of activated STAT (PIAS) and SOCS proteins are negative regulators of the JAK/STAT signaling pathway (Gao et al., 2018). Among SOCS proteins, SOCS3 is a major regulator of JAK/STAT signaling (Chen et al., 2000). Also, SOCS3 is a host protein that can be employed by viruses. SOCS3 can be induced by various viruses, including NDV, DHAV-1, HIV-1 and Enterovirus 71 (EV71) (Akhtar et al., 2010; Wang et al., 2019a; Xie et al., 2019; Gao et al., 2020). SOCS3 also inhibits or promotes viral replication when acted upon by small RNA molecules (Ma et al., 2018; Wang et al., 2019b; Duan et al., 2020). Furthermore, SOCS3 inhibits the catalytic activity of JAK2 by occupying the receptor or blocking substrate association. SOCS3 uses a short motif (the kinase- inhibitory region, KIR) to restrain signaling transmission by directly inhibiting the catalytic activity of JAKs. Moreover, SOCS3, through binding to gp130, inhibits STAT3 phosphorylation, and it also regulates the response to cytokines and growth factors (Nicholson et al., 2000; Yoshimura et al., 2007; Gao et al., 2018). Studies indicate that the tyrosine phosphorylation of SOCS3 accelerates the degradation of SOCS3 protein, thereby regulating feedback inhibition of JAK/STAT signaling (Haan et al., 2003; Ke and Liu, 2003).

The JAK/STAT pathway constitutes the fulcrum in many important cellular processes, including growth, differentiation, proliferation, and immune functions (Coskun et al., 2013). The binding of a cytokine to its cell-surface receptor results in receptor dimerization, resulting in the activation of JAKs *via* cross-phosphorylation. Specific tyrosine residues on the receptor are then phosphorylated by activated JAKs and serve as docking sites for a family of latent cytoplasmic transcription factors known as STATs. STATs are phosphorylated by JAKs, then dimerize and subsequently leave the receptor and translocate to the nucleus, where they activate gene transcription (Ke and Liu, 2003; Gao et al., 2018). In addition, JKA proteins can bind to the multichain receptor, such as the IL-2R family (*IL-2*, *IL-4*, *IL-7*, *IL-9*), the IL-3R family (*IL-3*, *IL-5*), IL-6R family (*IL-6*, *IL-11*, *IL-12*), and IFN-R family (*IFNs*, *IL-10*, *IL-19*, *IL-20*) (Coskun et al., 2013). Genetic knockout studies have shown that JAKs and STATs have highly specific functions in controlling various immune responses (Ke and Liu, 2003). The mice experimental study showed that JAK- or STAT- deficiency is lethal or immunodeficiency (Coskun et al., 2013). Viruses can evade the host immune system by inactivating different adaptors of the IFN-activated JAK/STAT signaling pathway (Ke and Liu, 2003). Under normal conditions, *STAT3* is low-expressed or not expressed in the signaling pathway. However, *STAT3* is activated when the host is stimulated. It was found that overactivated STAT3 protein promotes viral replication (Koeberlein et al., 2010; Okemoto et al., 2013).

ALV infection can result immunological tolerance, intermittent viremia, and persistent viremia (Dai et al., 2019; Yu et al., 2019). In the present study, we found that when the amount of ALV-J virus increased, the expression of *JAK2* and *STAT3* was also increased, indicating that *JAK2* and *STAT3* may have a positive effect on ALV-J persistent infection. Furthermore, when *SOCS3* was co-transfected with si-*JAK2* or si-*STAT3*, *SOCS3* affected JAK2 or STAT3 and promoted ALV-J virus replication. In addition, we also interfered with the expression of *JAK2* and *STAT3*, and found that both reduced *SOCS3* expression. These results verified that *SOCS3* is a negative regulator in JAK/STAT signaling pathway.

Interferon, inflammatory factors, and interferon-stimulating genes play an important role in the host’s innate immune response to antiviral infection. After overexpression of *SOCS3*, NDV, DHAV-1 and Hepatitis C virus (HCV) can reduce interferon and interferon-stimulated genes to evade IFN-mediated antiviral responses (Collins et al., 2014; Wang et al., 2019a; Xie et al., 2019). However, in this study, overexpression of *SOCS3* promoted the expression of those genes, while knockdown of *SOCS3* inhibited the expression of those genes, which may result from the ALV-J infection. Compared with other viruses, the clinicopathological changes of ALV-J virus infection require a longer period to appear under certain factors. Besides, some individuals may even be infected for life without disease clinicopathological changes, suggesting that ALV-J infection may be more complex than previously thought.

Altogether, these results suggest that SOCS3 promotes ALV-J replication *via* inhibiting JAK2/STAT3 phosphorylation. We conclude that *SOCS3* is an important negative regulator of the chicken innate immune signaling pathway. The finding was representing a substantial increase in our understanding of the mechanisms of persistent ALV-J infection.

## Data Availability Statement

The original contributions presented in the study are included in the article/**Supplementary Material**. Further inquiries can be directed to the corresponding author.

## Ethics Statement

The animal study was reviewed and approved by the Institutional Animal Care and Use Committee of South China Agriculture University.

## Author Contributions

Conceptualization, GM. Data curation, HF. Formal analysis, GM and HF. Funding acquisition, QN and XZ. Investigation, MX and LL. Methodology, GM and HF. Project administration, XZ. Resources, XZ. Software, QZ, MX, ZZ, and LL. Supervision QN and XZ. Validation, GM and BH. Visualization, QZ and HF. Writing, original draft, GM. Writing, review, and editing, BH and MS. All authors contributed to the article and approved the submitted version.

## Funding

This work was supported by the National Natural Science Foundation of China (31970540) and the China Agriculture Research System of MOF and MARA (CARS-41).

## Conflict of Interest

The authors declare that the research was conducted in the absence of any commercial or financial relationships that could be construed as a potential conflict of interest.

## Publisher’s Note

All claims expressed in this article are solely those of the authors and do not necessarily represent those of their affiliated organizations, or those of the publisher, the editors and the reviewers. Any product that may be evaluated in this article, or claim that may be made by its manufacturer, is not guaranteed or endorsed by the publisher.

## Supplementary Material

Supplementary Figure 1 The efficiency of overexpression and knock-down of SOCS3, JAK2 and STAT3 in DF-1 cell. According to the manufacturer’s instructions, the DF-1 cells were transfected with pcDNA3.1-SOCS3 plasmid or siRNAs by Lipofectamine 3000 reagent (Invitrogen, USA). After 24 h and 48 h transfection, the expression of SOCS3 **(A, B)**, was measured by qRT-PCR. After 24 h transfection, the expression of JAK2 **(C)**, and STAT3 **(D)** was measured by qRT-PCR. These experiments were performed independently at least three times with similar results. Differences in data were evaluated by the Student’s *t*-test. The error bars are the standard error of the mean (SEMs) (**P* ≤ 0.05, ***P* ≤ 0.01, and ****P* ≤ 0.001).
