# Supplementary figures and images for: SOCS3 Promotes ALV-J Virus Replication via Inhibiting JAK2/STAT3 Phosphorylation During Infection
Source: Front Cell Infect Microbiol. 2021 Sep 10;11:748795. doi: 10.3389/fcimb.2021.748795 (PMC8461107; doi:10.3389/fcimb.2021.748795)

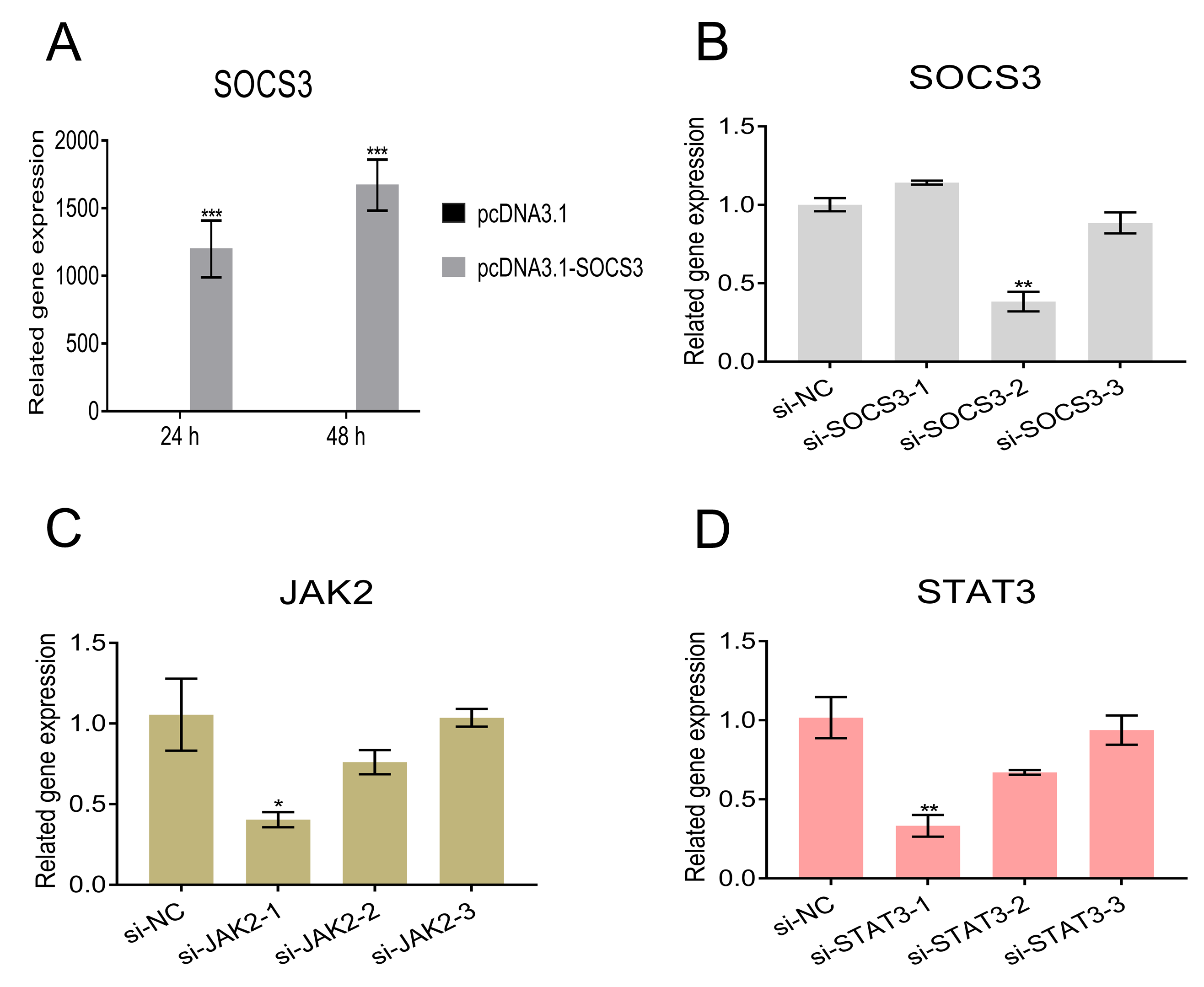

Supplement: Supplementary Figure 1 — The efficiency of overexpression and knock-down of SOCS3, JAK2 and STAT3 in DF-1 cell. According to the manufacturer’s instructions, the DF-1 cells were transfected with pcDNA3.1-SOCS3 plasmid or siRNAs by Lipofectamine 3000 reagent (Invitrogen, USA). After 24 h and 48 h transfection, the expression of SOCS3 (A, B), was measured by qRT-PCR. After 24 h transfection, the expression of JAK2 (C), and STAT3 (D) was measured by qRT-PCR. These experiments were performed independently at least three times with similar results. Differences in data were evaluated by the Student’s t-test. The error bars are the standard error of the mean (SEMs) (*P ≤ 0.05, **P ≤ 0.01, and ***P ≤ 0.001). [file Image_1.tif]
