# Supplementary material for: SOCS3 Promotes ALV-J Virus Replication via Inhibiting JAK2/STAT3 Phosphorylation During Infection
Source: Front Cell Infect Microbiol. 2021 Sep 10;11:748795. doi: 10.3389/fcimb.2021.748795 (PMC8461107; doi:10.3389/fcimb.2021.748795)
Supplement: Supplementary file 2 [file Table_1.docx]

**TABLE S1.** Primer information

| Name | Primer sequences (5’ to 3’) | Temperature (°C) |
| --- | --- | --- |
| *gp85* | TGTGTGCGTGGTTATTATTTC  AATGGCGAGGTCGCTGACTGC | 60 |
| *SOCS3* | CCAGCGGCACTTCTTCAC  CAGGTGCCCGTTGACAGT | 60 |
| *JAK2* | ATGGCACAAGCAGAGCAT  AGAACAGCCATTCCAAGG | 60 |
| *STAT3* | TGCCAGATGCCTAATGCC  AGTTCACACCTGGCCCTA | 60 |
| *IFNα* | GACAGCCAACGCCAAAGC  GTCGCTGCTGTCCAAGCATT | 60 |
| *IFNβ* | GCCCACACACTCCAAAACACTG  TTGATGCTGAGGTGAGCGTTG | 60 |
| *IL-6* | AAATCCCTCCTCGCCAATCT  CCCTCACGGTCTTCTCCATAAA | 60 |
| *TNFα* | AGCAGGGCTGACACGGAT  TGTTGGCATAGGCTGTCCTG | 60 |
| *CH25H* | AATCCAGCCGCAGAGCTATC  CAGCTCTGGAGCTATCACCG | 60 |
| *MX1* | AAGCCTGAGCATGAGCAGAA  TCTCAGGCTGTCAACAAGATCAA | 60 |
| *OASL* | CAGCGCCTGTACACCGAG  GGTTCTCCAGCTCCTTGGTC | 60 |
| *ZAP* | TTGATTCGGCGCCGCCTCTCTAC  ACTGGCCGTGGTCATTCTTC | 60 |
| *GAPDH* | GAACATCATCCCAGCGTCCA  CGGCAGGTCAGGTCAACAAC | 60 |
